# Developments of Key Data Element of Thalassemia Information Management Systems for Iran

**Published:** 2018-11

**Authors:** Leila KEIKHA, Seide Sedigheh SEYED FARAJOLLAH, Reza SAFDARI, Marjan GHAZI SAEEDI

**Affiliations:** Dept. of Health Information Management, School of Allied Medical Sciences, Tehran University of Medical Sciences, Tehran, Iran

## Dear Editor-in-Chief

In Iran, more than 2-3 million people are thalassemia major carrier which from them 25000 people are suffering from the disease (1, 2). This comprises 4% of the Iranian population (3). Thalassemia major is a kind of beta thalassemia that is most prevalent in Mazandaran, Gilan, Hormozgan, Khuzestan, Kohgiluyeh, Fars, Bushehr, Sistan and Baluchestan, Kerman, and Isfahan (4, 5). The existence of appropriate, updated, and integrated information about the patients and carriers of this disease is a practical tool for managing, preventing, and reducing the effects that arise from this disease (6). In Iran, a number of clinical minimum data sets have been introduced so far in different fields; hemodialysis disease (7), burns (8) and anesthesia for cesarean delivery (9) are the newest datasets that published. The aim of this study was to create a minimum data set for managing thalassemia throughout various information systems including electronic health record and registries.

In this descriptive cross-sectional study, conducted in 2016, the development of minimum datasets was done by extracting data from hospital medical records based on the D55–D59 category of International Classification of Diseases 10th revision (ICD-10). Then, the final questionnaire designed according to the checklist provided by American health information management Association (AHIMA) to develop the minimum data set for EHR in 2006. Finally, by using the Delphi method, the final questionnaire by available sampling sent to 12 specialists including health information management specialists, genetics specialist, hematologists, and general practitioners, so that their views would be collected on the questionnaire. As a result of the Delphi study, data elements at 11 categories were determined.

Administrative Data including patient full name, father’s full name, mother’s full name, identifier number, sex, religion, birth date, marital status, education level, occupation, ethnic groups, occupation, place of work, address, blood group, RH Type, Consent, brought or referred by, patient education, comorbidity, and authenticator/signature were determined.

Encounter data consist of primary diagnosis, final diagnosis, date/time of admission, kind of admission, admitting physician, admitted from, body mass index, history of hospitalization, the number of hospitalization, length of stay, transfusion information, ward name, date/time of discharge, death data, and authentication/signature after Delphi technique were taken into account.

Treatment Plan Data contain Inpatient order, Blood reserve order, Blood transfusion order, consultations orders, lab-tests orders, radiography orders, medications orders, dietary type, order for the following, discharge order, transfer order, time/date of the order, Chelation therapy, surgery order, and authentication/signature.

Provider data including provider full name, specialty, provider address, provider telephone, provider role, unique provider identifiers, and authenticator/signature were considered.

Examination data were following as; date/time of transfusion, Interval between transfusion, volume of transfused blood, warm blood, transfuse products, patient blood culture, bag culture, complication after transfusion, requested blood volume, iron overload, resistant to hepatitis c, non-resistant to hepatitis c, Desferal pomp type, vital signs before transfusion, vital signs in transfusion, vital signs after transfusion, pre-operative examination, examination during operation, examination post-operative, injectable drugs and solutions during transfusion, adverse reaction to blood transfusion, Desferal side effects, history of unexpected antibody in serum and authentication/signature.

History data were detailed as presenting symptom, past disease history, history of blood transfusion reaction, current drug therapy & other addictions, drug or food allergy, family history and authentication/signature.

Diagnostic test data contain laboratory test, radiology and radiography report, genetic test, pathology test and authenticator/signature.

Event data were considered as the reason for the visit, chief complaint, patient visit information, clinical progress note, and authenticator/signature.

Insurance data including authenticator/signature, type of insurance, insurance ID, accident insurance, the total cost of operations, therapies, surgeon, specimen, drugs and special care for insurance data were considered.

Follow-up data consists of date/time of follow-up, follow-up visit data, Iron load, chelation associated follow-up, liver function, cardiac function, endocrine function, bone complication, infections, dentition, rehabilitation and Signature/authenticator. Blood product concludes blood type, unit, number, blood group and RH of requested product, blood pack serial number, expired date, and authenticate.

This minimum data set is not only applicable in Iran, but it has potential to use in the national and international level. All of the above mentioned will reduce the cost of thalassemia patients. Strategic plan for development infrastructures, standards, and communication tools for integrating such subsystems to electronic health record system (EHRS) is necessary for future research.
